# Oxidized lipids keep heat shock chaperones busy: new insights on the deficiencies of tumour-associated dendritic cells

**DOI:** 10.1186/s40425-018-0373-3

**Published:** 2018-06-20

**Authors:** Paula Nunes-Hasler

**Affiliations:** Department of Cell Physiology and Metabolism, University of Geneva, Faculty of Medicine, Rue Michel-Servet 1, 1211 Geneva, Switzerland

**Keywords:** Heat-shock protein, Hspa1a, Hsp72m, Lipid bodies, Antigen presentation, Cross-presentation

## Abstract

In a recent publication in *Nature Communications* the group of Dr. Dmitry Gabrilovich takes us one step closer to understanding why lipid accumulation impairs the function of tumour-associated dendritic cells (DCs). In this study, the authors present two surprising and significant findings. First, they show that in mouse DCs oxidized lipids function as a sink that traps the heat shock chaperone HSP70, a molecular target of emerging anti-cancer strategies. Secondly, they find that HSP70 in turn regulates the trafficking of peptide-loaded major histocompatibility complex class I (pMHC-I) molecules, a complex that triggers the proliferation of cancer-killing T cells. These observations are discussed briefly in the context of lipid droplet function and pMHC-I trafficking in tumour-associated DCs, as well as HSP70’s pleiotropic and incompletely understood roles - and what they mean for future cancer therapy designs.

## Main text

Dendritic cells (DCs) are antigen-presenting immune cells pivotal to anti-cancer immunity because of their unique ability to capture tumour-associated antigens and use them to activate cancer-killing cytolytic T cells. This process, termed cross-presentation, involves loading tumour-derived peptides onto major histocompatibility complex I (MHC-I) molecules. These peptide-MHC-I complexes (pMHC-I) then traffic to the cell surface, where together with co-stimulatory molecules they trigger the proliferation and differentiation CD8^+^ T cells into cancer-specific cytotoxic T cells. Patients whose tumours harbour a greater number of DCs have a better prognosis [1], yet clearly at some point this system breaks down. Indeed, it is now well-recognized that DCs from tumour-bearing hosts have a blunted cross-presentation activity [1]. Exactly why this is so is not fully understood, but a number of factors in the tumour microenvironment have been postulated to play a role including hypoxia, pH, and higher levels of adenosine, lactate and immunosuppressive factors such as IL-10 and PD-L1 [1].

Notably in 2010 the Gabrilovich group observed an abnormal accumulation of lipid droplets (also known as lipid bodies) within tumour-associated DCs, which when inhibited, restored cross-presentation [2]. Lipid droplets are organelles classically viewed as lipid storage sites, and although relatively little is known about their other physiological roles in DCs, this observation was surprising given that previous work indicated that lipid droplets promote rather than inhibit cross-presentation [3]. Indeed saponin-based adjuvants were suggested to increase cross-presentation because of their ability to *increase* DC lipid droplets [4]. However, the same group then identified oxidized lipids as specifically causing blunted cross-presentation, as the effect was not seen with non-oxidized lipids [5]. This observation helps explain why generalized lipid accumulation may be beneficial in other contexts, yet exactly how lipid droplets can influence antigen processing or cross-presentation remained unclear.

In their most recent paper published in *Nature Communications, Veglia* et al. now provide an unexpected potential mechanism involving the heat-shock-induced chaperone heat-shock protein 70 (HSP70) that might explain how oxidized lipid accumulation can lead to defective cross-presentation [6]. By combining lipidomics and molecular dynamic simulations the authors show that oxidatively truncated (ox-tr) triacylglycerides accumulate in large lipid droplets of tumour-associated mouse DCs, and that these highly electrophilic species are predicted to preferentially occupy the droplet surface where they can directly access cytosolic proteins. These ox-tr lipids contain reactive functional groups that were capable of mediating a covalent attachment to HSP70, and lipid droplets of DCs exposed to tumour extracts but not of controls strongly accumulated HSP70. These data suggest that not all lipid droplets are created equal, and that these *oxidized* lipid droplets essentially serve as a sink that traps cytosolic HSP70, preventing it from performing other functions.

This of course begs the next question, which is then how can HSP70 influence cross-presentation? Gabrilovich’s team begins to answer this with an astonishing observation that either inhibiting or depleting HSP70 leads to a re-routing of pMHC-I complexes, which rather than reaching the plasma membrane, are sent to lysosomes instead. Yet the remaining question of how HSP70 can regulate pMHC-I trafficking is perhaps the most intriguing, considering trafficking control is hardly a well-established role for the ubiquitous chaperone. HSP70 is best known for its protective role during cellular stress where it binds hydrophobic patches of unfolded proteins, helping them to refold and preventing their aggregation [7]. Cytoprotection is thought to be a key reason why HSP70 is overexpressed in many different types of cancer, and in part why its inhibition has shown promise as an anti-cancer therapy [7]. However a myriad of other functions with immunomodulatory consequences have now also been ascribed to HSP70 [7]. It can be secreted either in soluble form or associated to exosomes and can induce cytokine secretion alone or in concert with bound clients. It promotes antigen presentation of bound clients, and has even been proposed to do so by helping antigens directly cross membranes, in a manner similar to the HIV TAT peptide. Extracellular HSP70 also binds scavenger receptors such as CD91 and LOX-1 leading to their activation and internalization, it can enhance TLR signalling, and can alone induce generalized increases in endocytosis [7]. Recent advances on the regulation of MHC-I trafficking include a role for TLR signalling which alters the phosphorylation of SNARE proteins to redirect the fusion of MHC-I bearing vesicles [8]. Thus, whether TLR signalling is implicated in HSP70 regulation of pMHC-I would be interesting to explore. On the other hand, clues that cytosolic HSP70 may control trafficking through direct binding have been reported for the AQP2 channel, where phosphorylation-dependent HSP70 binding of the channel’s cytosolic tail recruits ubiquitin ligases to help target the channel for destruction [9, 10] yet such a mechanism for pMHC-I would be predicted to have the opposite effect of that observed by *Veglia* et al.

Thus, a primary outstanding issue is whether it is intracellular or extracellular HSP70 that controls pMHC-I trafficking. It is curious to note that effects of HSP70 modulation were restricted to pMHC-I complexes, as the same was not observed when total MHC-I molecules were probed. Moreover, oxidized lipids influenced only the surface levels of pMHC-I bearing exogenous but not endogenous peptides [5]. Taken together these data raise the intriguing possibility that HSP70 not only regulates pMHC-I trafficking, but that it somehow specifically senses exogenous pMHC-I complexes. How DCs differentiate between exogenous and endogenous peptides loaded onto MHC-I is one of the great mysteries of DC biology. Understanding this subject is of paramount importance if we are to successfully manipulate DCs into specifically generating anti-tumour responses without running the risk of stimulating undesired autoimmune reactions.

Overall, the research of Veglia and colleagues raises caution in employing HSP70 inhibitors as anti-cancer agents, as their use may prevent the initiation of anti-tumour immunity by DCs. On the other hand, deciphering which specific HSP70 forms are responsible for regulating pMHC-I complexes on DCs could help define whether tailoring such strategies to selectively target different HSP70 species could bypass these negative effects. It should be noted that the study was performed using mouse DCs and confirmation that similar effects occur in human cells would be beneficial. Additionally, this work suggests that lipid oxidation in the tumour microenvironment is a key factor in suppressing the anti-tumour capabilities of DCs, and favours therapeutic strategies including the administration of antioxidants such as vitamin E [6]. Thus, another possibility is to co-administer HSP70 inhibitors together with antioxidants. One may also envisage that lipid depletion of tumour extracts or the addition of antioxidants could be beneficial during DC priming for the preparation of DC-based vaccines. Finally, it could be informative to re-examine whether using saponin-based adjuvants is counterproductive by increasing ox-tr lipid uptake, or conversely whether the promotion of non-oxidized lipid accumulation could serve to counteract the suppressive effect of oxidized lipids. Clearly more work is needed to gain a deeper understanding on the precise roles of lipid droplets, lipid-modifying agents and chaperones in regulating DC functions, as these may have direct effects on the efficacy of future therapy designs.

## Abbreviations

AQP2: Aquaporin-2
DC: Dendritic cell
HIV: Human immunodeficiency virus
HSP70: Heat-shock protein 70 (also called Hspa1a/Hsp72)
IL-10: Interleukin 10
LOX-1: Lectin-like oxidized low-density lipoprotein receptor 1
MHC-I: Major histocompatibility complex class I
Ox-tr: Oxidatively truncated
PD-L1: Programmed death ligand 1
pMHC-I: Peptide-loaded major histocompatibility complex class I
SNARE: Soluble N-ethylmaleimide-sensitive factor attachment protein receptor
TAT: Transactivator of transcription
TLR: Toll-like receptor
